# Bromethyl

**Published:** 1891-01-24

**Authors:** 


### THERAPEUTICS.

### BROMETHYL.

Dr. Falk, after digesting the entire literature of the subject, comes to the conclusion that Bromethyl approaches nearer than any other body to the character of an ideal anæsthetic. The initial excitement is far less than in the case of chloroform. Anæsthesia supervenes very rapidly, *i.e.*, in fifteen to twenty seconds after the administration is begun, analgesia precedes loss of consciousness, the return to complete consciousness and power is rapid, and unpleasant after-effects are rare. Haffter, who has studied its employment and phenomena more closely than anyone, says that a creeping sensation is experienced by the patient, commencing in the extremities and proceeding upwards to the head, the inspirations become deep and slow, until at about the sixth or eighth second they are suspended for a few moments, but without any sense of suffocation, after which they begin again as before; the pupils dilate, the cheeks and forehead flush, the skin perspires, sensation is lost, and consciousness later: during narcosis the dreams are pleasant and, in perhaps a third of the cases, erotic; on awaking recovery is almost instantaneous and complete. Some observers notice no excitement whatever, others do so rarely, and some few have seen it occur on awaking. The heart's action appears to be slightly accelerated, and in a few cases palpitation has been observed. Vomiting is very rare during the narcosis, but occasionally occurs afterwards; this, however, is far less frequent than with chloroform, and is less inconvenient and dangerous from the patient being already fully conscious. Experiments on animals leave no doubt that in health, death is always due to paralysis of the diaphragm, the heart continuing to beat for some time longer, though when this organ is diseased, especially when weak and fatty, and still more if there be co-existing disease of the lungs, the heart's action may be suddenly arrested. Indeed, we may lay it down as a general rule in practical therapeutics that drugs of whatever kind which are described, and from a physiological point of view correctly described, as acting solely on the respiratory centres should be given with the utmost caution in heart disease, since any impediment to the respiration must inevitably re-action the heart. Hitherto, Bromethyl has been little used except by dentists, but while nitrous oxide is certainly best adapted for their purposes, this anæsthetic seems to merit more general employment in surgery. We, ourselves, are convinced that chloroform, or chloroform followed by ether, as is our usual practice, is the most convenient and safest in obstetric operations. Montgomery's experience of Bromethyl in such cases does not appear favourable, though we have not as much information as we could wish. He states that the peculiar phosphorous odour of the breath could be perceived in that of the infant also, and that in twenty-nine cases one was stillborn, one died soon after in convulsions, and another on the third day cyanotic. Generally, ten to twenty grams only should be given (say ʒii to ʒiv fl.), and that all at once in a close-fitting mask. The cases in which convulsions occured were all those of epileptics, or persons suffering from renal disease, in whom the same phenomena had been observed previously; in fact they were unsuitable, and in one, reported by Dr. Marion Sims, the enormous quantity of 150 grams had been administered. The danger of chloroform is in concentration, that of Bromethyl in dilution. In operations, therefore, in which the patient has to be kept for a long time under the influence of the anæsthetic, and in obstetrical cases, where only partial, though long continued, anæthesia is desired, Bromethyl is positively out of place, and chloroform as clearly to be preferred; if, indeed, there be any option in the matter, Bromethyl should never be given in renal disease, in patients with weak and fatty hearts, or advanced disease of the lungs, or in persons prone to fainting.

